# Retraction: Long noncoding RNA LOC400043 (LINC02381) inhibits gastric cancer progression through regulating Wnt signaling pathway

**DOI:** 10.3389/fonc.2023.1298859

**Published:** 2023-12-05

**Authors:** Frontiers Editorial Office

The journal retracts the October 23, 2020, article cited above.

Following publication, concerns were raised regarding the integrity of the images in the published figures. Image duplication concerns were identified in Figure 6B.

The authors failed to provide a satisfactory explanation during the investigation, which was conducted in accordance with Frontiers’ policies. As a result, the data and conclusions of the article have been deemed unreliable and the article has been retracted.

The authors and their institution have remained unresponsive to multiple requests for the raw data for this study. Given extant concerns over the validity of the study, the article has been retracted.

This retraction was approved by the Chief Editors of Frontiers in Oncology and the Chief Executive Editor of Frontiers.

The authors disagreed with this retraction.

